# Smad1 promotes colorectal cancer cell migration through Ajuba transactivation

**DOI:** 10.18632/oncotarget.22780

**Published:** 2017-11-30

**Authors:** Daming Yang, Tieying Hou, Lei Li, Yimin Chu, Fengli Zhou, Ying Xu, Xinyu Hou, Huan Song, Kai Zhu, Zhaoyuan Hou, Haixia Peng, Hao Jia

**Affiliations:** ^1^ Digestive Endoscopy Center, Tongren Hospital, Shanghai Jiaotong University School of Medicine, Shanghai 200336, China; ^2^ Hongqiao Institute of Medicine, Tongren Hospital/Faculty of Basic Medicine, Shanghai Jiaotong University School of Medicine, Shanghai 200025, China; ^3^ Department of Clinical Laboratory, Guangdong General Hospital, Guangdong Academy of Medical Sciences, Guangzhou 510080, China; ^4^ Department of Thoracic Surgery, Lanling People's Hospital, Lanling County, Linyi 277700, China; ^5^ Shanghai Key Laboratory for Tumor Microenvironment and Inflammation, Department of Biochemistry & Molecular Cellular Biology, Shanghai Jiaotong University School of Medicine, Shanghai 200025, China; ^6^ Department of Gastroenterology, Ruijin Hospital, Shanghai Jiaotong University School of Medicine, Shanghai 200025, China; ^7^ Liver Cancer Institute, Zhongshan Hospital, Fudan University, Shanghai 200032, China

**Keywords:** Smad1, Ajuba, migration, colorectal cancer, Snail

## Abstract

SMAD family member 1 (Smad1) have been involved in metastatic progression of many cancer types. However, the detailed molecular signalling pathway underlying the regulatory link between Smad1 and metastasis remains elusive. Here, we demonstrate that Smad1 promotes migration of colorectal cancer (CRC) cells by inducing Snail and Ajuba expression simultaneously, but no apparent effect on Twist1 expression. Remarkably, E-cadherin, the best known Snail/Ajuba target gene is downregulated by Smad1 expression. Further, depletion of Ajuba in HCT116 cells significantly dampens the cell migration capability induced by Smad1 overexpression, suggesting that Ajuba is required for Smad1 to induce cell migration. Moreover, clinical analysis shows a significant positive correlation between the level of Smad1 and Ajuba in CRC samples. Together, our data provides the first evidence of the regulatory network of Smad1/Snail/Ajuba axis in CRC migration, suggesting that Smad1 and Ajuba are potential new therapeutic targets and prognostic factors for CRC.

## INTRODUCTION

SMA and mother against decapentaplegic (MAD)-related proteins (SMADs) are intracellular components of TGF-ß signaling pathway [2]. So far, there are eight members in SMAD family, designated as Smad1-Smad8 respectively. According to the structure and function, SMADs are further divided into subgroups: receptor-regulated SMADs (R-Smad including Smads1/2/3/5/8), a common SMAD (Co-Smad containing Smad4 only), and inhibitory SMADs (I-Smads) [3, 4, 19, 21]. For R-Smads, Smad1/5/8 transduce bone morphogenetic protein (BMP) signals, whereas Smad2/3 generally mediate TGFβ signaling pathway [12, 17].

Dysregulation of Smad functions are linked to various types of developmental defects and diseases. For example, Smad1 can be induced by many tumor-stimulating cytokines such as the bone morphogenetic protein 2 (BMP2) and TNFα and plays important roles in cell invasion and metastasis [10, 20, 23]. Additionally, Smad1 is upregulated by B7-H3 via PI3K-Akt pathway and promotes Epithelial-Mesenchymal Transition (EMT) process in the colorectal cancer cells [9]. Moreover, Smad1 is identified as a target of miR-26b-5p in hepatocellular carcinoma metastasis and EMT [22]. These observations suggest a link between Smad1 and EMT. However, the detailed molecular signaling pathway underlying the regulatory link between Smad1 and cell migration and EMT remains elusive.

The Snail transcription factor is a master regulator of EMT and promotes cell migration during normal development and tumor metastases. Previous studies showed transcriptional repression and EMT induction by Snail requires recruiting Ajuba as a co-repressor [1]. Ajuba is a scaffold protein, belonging to the Zyxin/Ajuba family characterized by two or three tandem C-terminal LIM domains and a unique N-terminal region designated as the preLIM region [5]. Ajuba is involved in many cellular processes such as cell-cell adhesion, gene transcription, cell proliferation, cell migration, and cytokinesis [1, 6]. Several studies also suggest that Ajuba promotes EMT in colorectal cancer [15]. Most recently, we found that Ajuba is markedly upregulated in CRC and promotes CRC development by inhibiting cell apoptosis [8]. However, how the expression of Ajuba is regulated in CRC cells remain undefined.

In the present study, we demonstrate that Smad1 induces the expression of Ajuba and Snail to promote cell migration in colorectal cancer cells. Clinically, the level of Smad1 in CRC specimens is positively correlated with the levels of Ajuba. The present work provides the first evidence of the regulatory network of Smad1-Ajuba axis in CRC, suggesting that Smad1 and Ajuba are potential new therapeutic targets and prognostic factors for CRC.

## RESULTS

### Smad1 promotes cell migration of colorectal cancer cells

**Figure 1:**
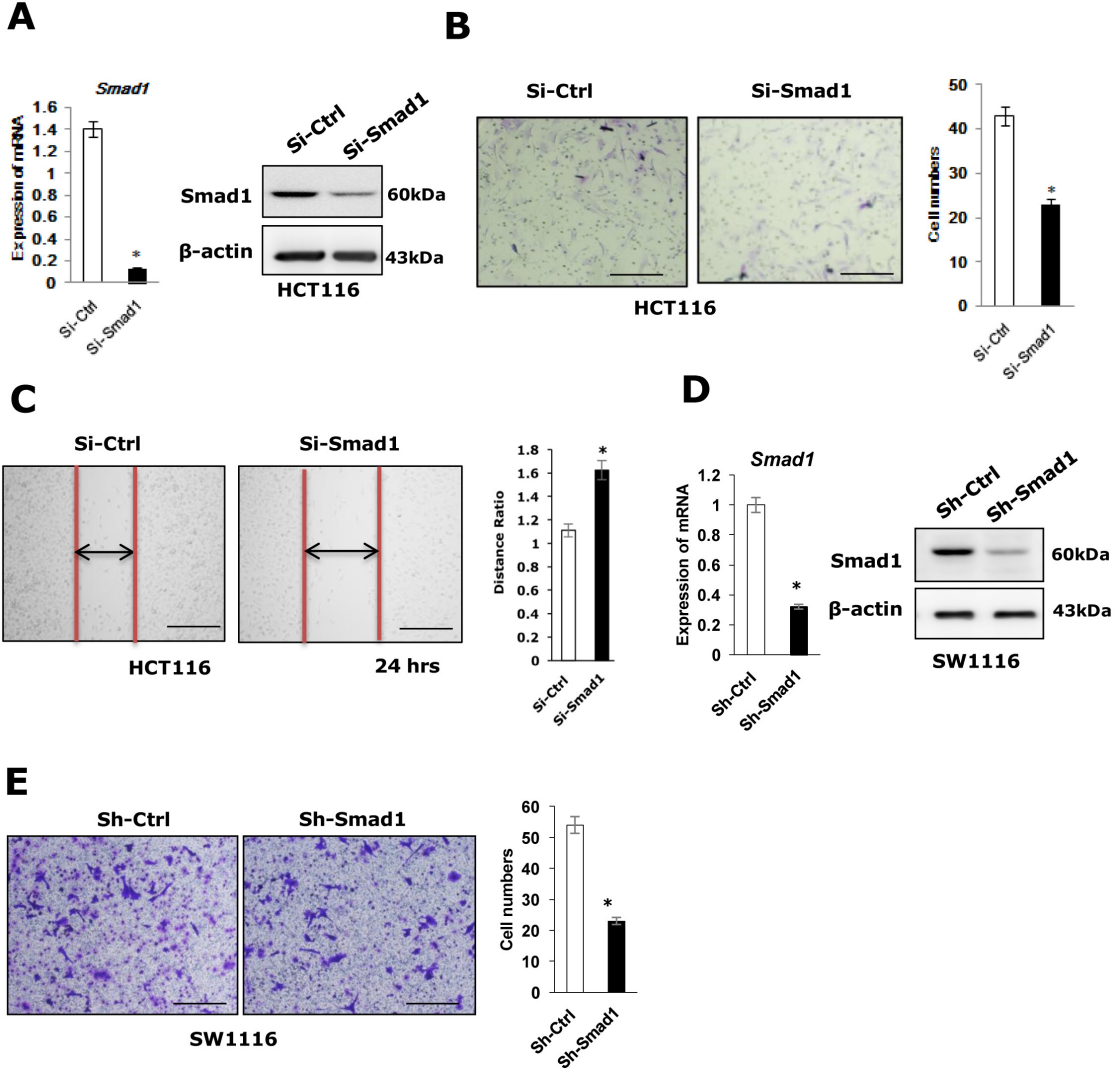
Knocking-down of Smad1 decreases CRC cell migration **(A)** Left panel: The mRNA expression of Smad1 in HCT116 cell lines (Si-Ctrl, Si-Smad1) were shown. Data were shown as mean± S.D. from three independent experiments, ^*^p<0.05(n=3). Right panel: The protein expression of Smad1 in HCT116 cell lines (Si-Ctrl, Si-Smad1) were shown. **(B)** Left panel: The migration ability of HCT116 cells was measured by transwell assay, the assays were repeated three times in triplicate. Right panel: Statistics were showed with bar graph. Data were shown as mean± S.D. from three independent experiments, ^*^p<0.05(n=3). **(C)** Left panel: The motility of HCT116 cells was determined by scratching assay and statistics were showed with bar graph. Right panel: Data were shown as mean± S.D. from three independent experiments, ^*^p<0.05(n=3). **(D)** Left panel: The mRNA expression of Smad1 in SW-1116 cell lines (Sh-Ctrl, Sh-Smad1) were shown. Data were shown as mean± S.D. from three independent experiments, ^*^p<0.05(n=3). Right panel: The protein expression of Smad1 in SW-1116 cell lines (Sh-Ctrl, Sh-Smad1) were shown. **(E)** Left panel: The migration ability of SW-1116 cells was measured by transwell. This assays were repeated three times in triplicate. Right panel: Statistics were showed with bar graph. Data were shown as mean± S.D. from three independent experiments, ^*^p<0.05(n=3).

**Figure 2:**
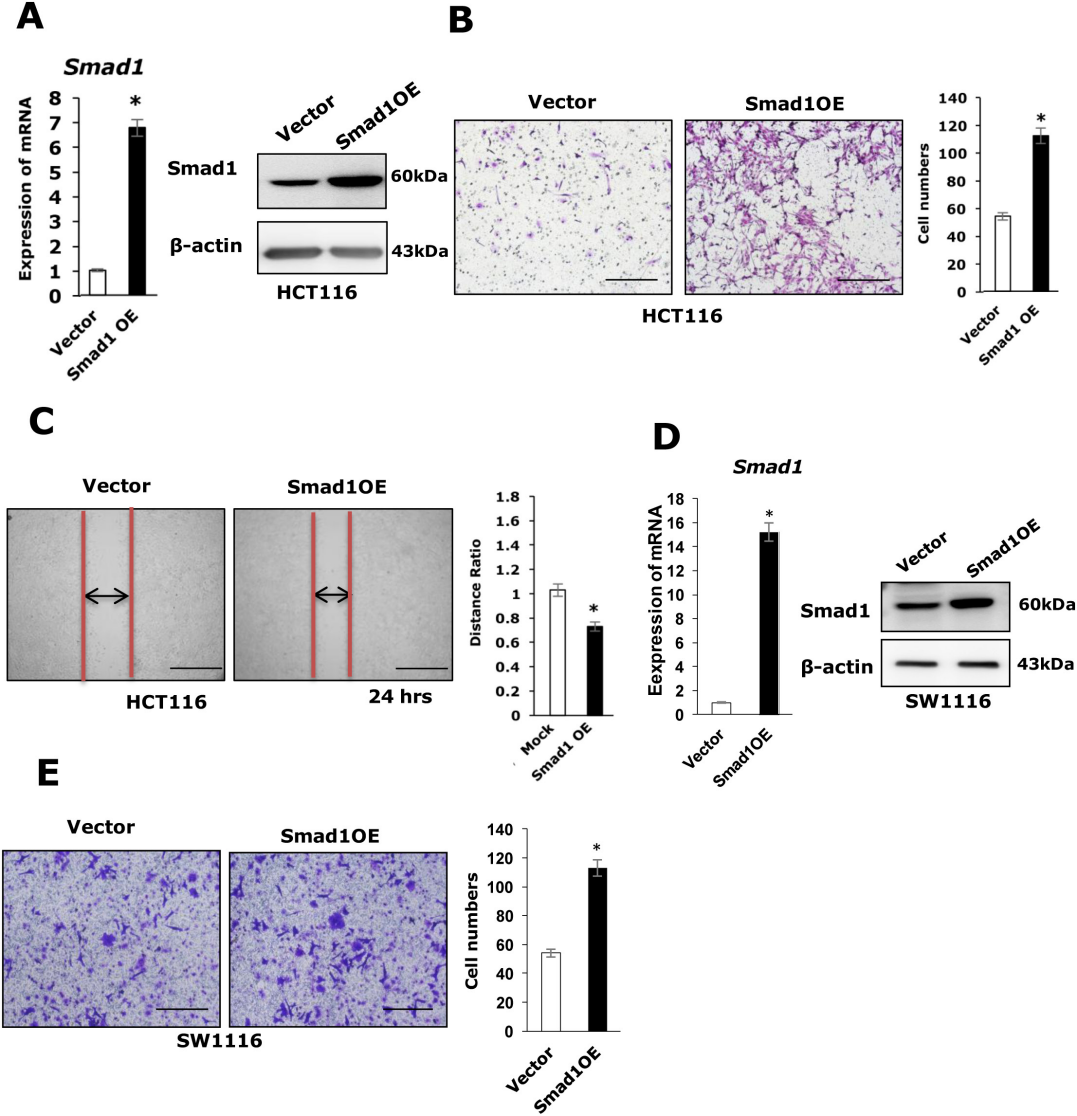
Smad1 promotes CRC cell migration **(A)** Left panel: The mRNA expression of Smad1 in HCT116 cell lines (Vector, Smad1 OE) were shown. Data were shown as mean± S.D. from three independent experiments, ^*^p<0.05(n=3). Right panel: The protein expression of Smad1 in HCT116 cell lines (Vector, Smad1 OE) were shown. **(B)** Left panel: The migration ability of HCT116 cells was measured by transwell assay, the assays were repeated three times in triplicate. Right panel: Statistics were showed with bar graph. Data were shown as mean± S.D. from three independent experiments, ^*^p<0.05(n=3). **(C)** Left panel: The motility of HCT116 cells was determined by scratching assay and statistics were showed with bar graph. Right panel: Data were shown as mean± S.D. from three independent experiments, ^*^p<0.05(n=3). **(D)** Left panel: The mRNA expression of Smad1 in SW-1116 cell lines (Vector, Smad1 OE) were shown. Data were shown as mean± S.D. from three independent experiments, ^*^p<0.05(n=3). Right panel: The protein expression of Smad1 in SW-1116 cell lines (Vector, Smad1 OE) were shown. **(E)** Left panel: The migration ability of SW-1116 cells was measured by transwell. This assays were repeated three times in triplicate. Right panel: Statistics were showed with bar graph. Data were shown as mean± S.D. from three independent experiments, ^*^p<0.05(n=3).

To determine whether Smad1 regulates cell migration in colon cancers, Smad1 was depleted or overexpressed in HCT116/Sw1116 cells using Smad1 siRNA/shRNA or PCDNA3.0-Smad1-expressing plasmid, the protein and the mRNA level of Smad1 were examined by western-blot and qRT-PCR assays (Figures 1A, 1D, 2A, 2D, Supplementary Figure 1A-1D). The migratory capability of the resulting cells was examined by transwell and scratching assays. Depletion of Smad1 resulted in significantly reduced migratory capacities as compared with control cells (Figure 1B, 1C, 1E). In contrast, ectopic expression of Smad1 in HCT116/Sw1116 cells enhanced cell migration (Figure 2B, 2C, 2E). We next performed CCK-8 assays to examine the effect of Smad1 on the overall cell growth. Notably, depletion or stable expression of Smad1 in HCT116 cells was not affect the cell growth (Supplementary Figure 2). Together, these data indicate that Smad1 is essential for migratory capability of HCT116 cells.

### Ectopic expression of Smad1 induces Snail and Ajuba expression in HCT116 cells

**Figure 3:**
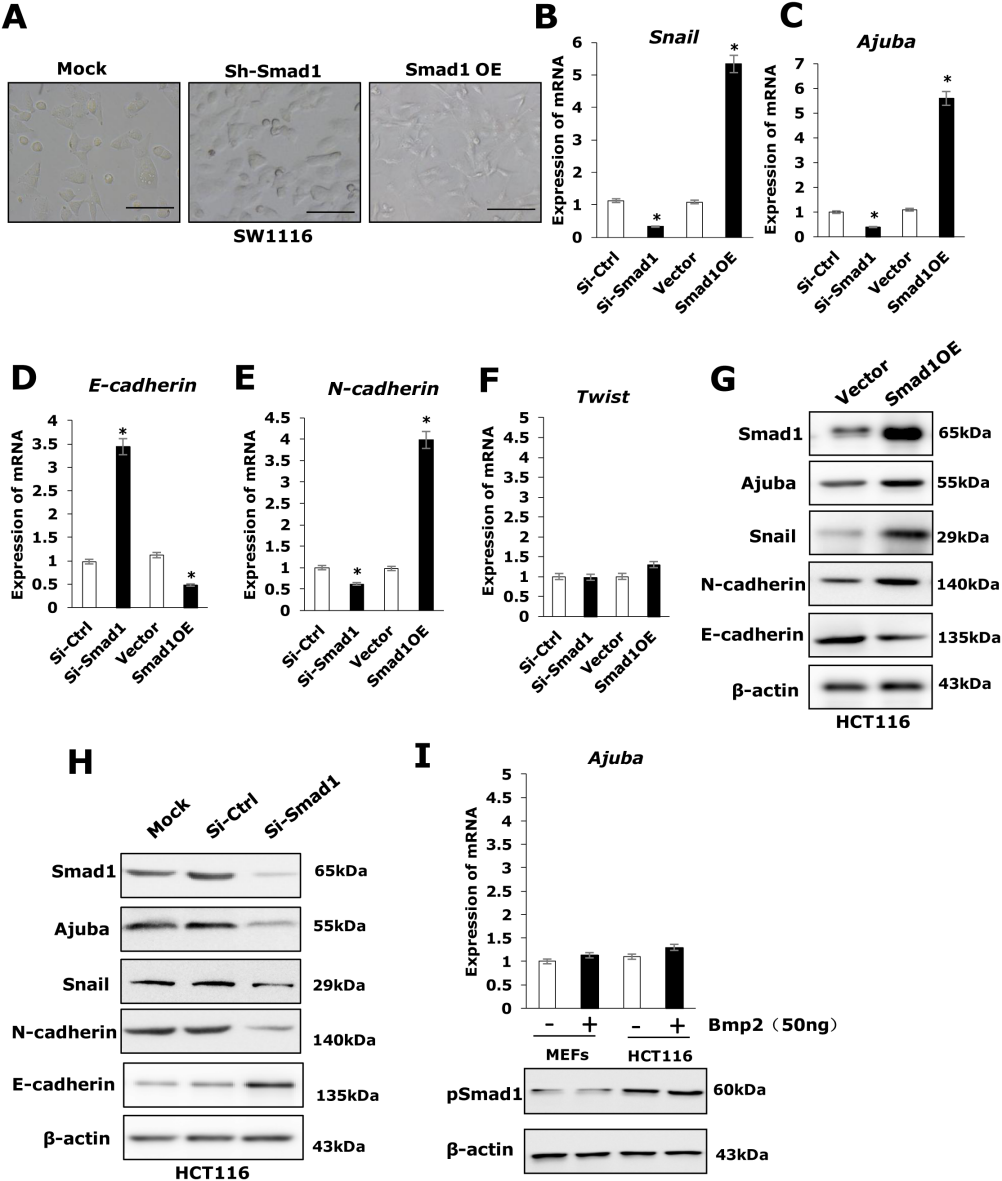
Smad1 promotes migration of HCT116 by enhancing EMT **(A)** The cellular morphology of three cell lines (SW1116-Mock, SW1116-shSmad1, and SW1116-Smad1) is shown. **(B)** The mRNA expression of epithelial and mesenchymal markers, Snail, as well as the transcription factors, was compared between HCT116 cell lines (Si-Ctrl, Si-Smad1, Vector, Smad1OE) by qRT-PCR. Data were shown as mean± S.D. from three independent experiments, ^*^p<0.05(n=3). **(C)** The mRNA expression of Ajuba was compared between HCT116 cell lines (Si-Ctrl, Si-Smad1, Vector, Smad1OE) by qRT-PCR. Data were shown as mean± S.D. from three independent experiments, ^*^p<0.05(n=3). **(D)** The expression of epithelial and mesenchymal markers, E-cadherin, as well as the transcription factors, was compared between HCT116 cell lines (Si-Ctrl, Si-Smad1, Vector, Smad1OE) by qRT-PCR. Data were shown as mean± S.D. from three independent experiments, ^*^p<0.05(n=3). **(E)** The expression of epithelial and mesenchymal markers, N-cadherin, as well as the transcription factors, was compared between HCT116 cell lines (Si-Ctrl, Si-Smad1, Vector, Smad1OE) by qRT-PCR. Data were shown as mean± S.D. from three independent experiments, ^*^p<0.05(n=3). **(F)** The expression of epithelial and mesenchymal markers, Twist, as well as the transcription factors, was compared between HCT116 cell lines (Si-Ctrl, Si-Smad1, Vector, Smad1OE) by qRT-PCR. Data were shown as mean± S.D. from three independent experiments, ^*^p<0.05(n=3). **(G-H)** Western blots show the EMT protein in HCT116 cells and the derivatives. **(I)** Up: Bmp2 did not induces Ajuba in MEFs and Hct116 cells. The cells were treated with 50 ng/ml Bmp2 or PBS. After12 hrs, the total RNAs were extracted from the resulting cells for qRT-PCR analysis. Data were shown as mean± S.D. from three independent experiments, ^*^p<0.05(n=3). Down: Western blots show the pSmad1 protein in HCT116 cells and MEFs.

We first examined the cellular morphology of three cell lines with different expression levels of Smad1 (SW1116-Mock, sh-Smad1, Smad1-OE). A distinct morphological difference was observed in those cell lines (Figure 3A). To determine if Smad1 is able to increase the expression of the known cell migration inducers such as Twist1, Snail and its cofactor Ajuba as well as EMT markers E-cadherin and N-cadherin, total RNAs and protein were extracted from HCT116-vector, HCT116-Smad1, Si-Smad1 cells or Si-Ctrl were prepared. qRT-PCR and Western-blot assays were performed to examine the expression of the chosen genes. Strikingly, ectopic expression of Smad1 markedly increased the mRNA and protein level of Snail and Ajuba, and depletion of Smad1 decreased the mRNA and protein level of Snail (Figure 3B, 3G) and Ajuba (Figure 3C, 3G, 3H and Supplementary Figure 1E, 1F), while the mRNA and protein level of Twist1 remained unchanged (Figure 3F, 3G, 3H and Supplementary Figure 1E, 1F). Remarkably, E-cadherin (Figure 3D, 3G, 3H and Supplementary Figure 1E, 1F), the best known Snail/Ajuba target gene was downregulated by Smad1 overexpression and was induced by Smad1 depletion. Conversely, the expression of N-cadherin showed opposite pattern to Snail and Ajuba (Figure 3F, 3G, 3H and Supplementary Figure 1E, 1F). To validate if the expression of Ajuba was affected by BMP2, we treated HCT116 cells and MEFs cells with bmp2 for 12 h and the total RNAs were extracted from the resulting cells for qRT-PCR analysis. Surprisingly, the expression of Ajuba was not apparently affected by BMP2 treatment (Figure 3I and Supplementary Figure 1G). We also had added the p-Smad1 as the positive control to prove the Ajuba was not regulated by Bmp2 (Figure 3I). Collectively, these observations suggest that Smad1 regulate EMT by transactivating Snail/Ajuba expression in HCT116 cells.

### Ajuba is required for Smad1 to increase cell migration in HCT116 cells

**Figure 4:**
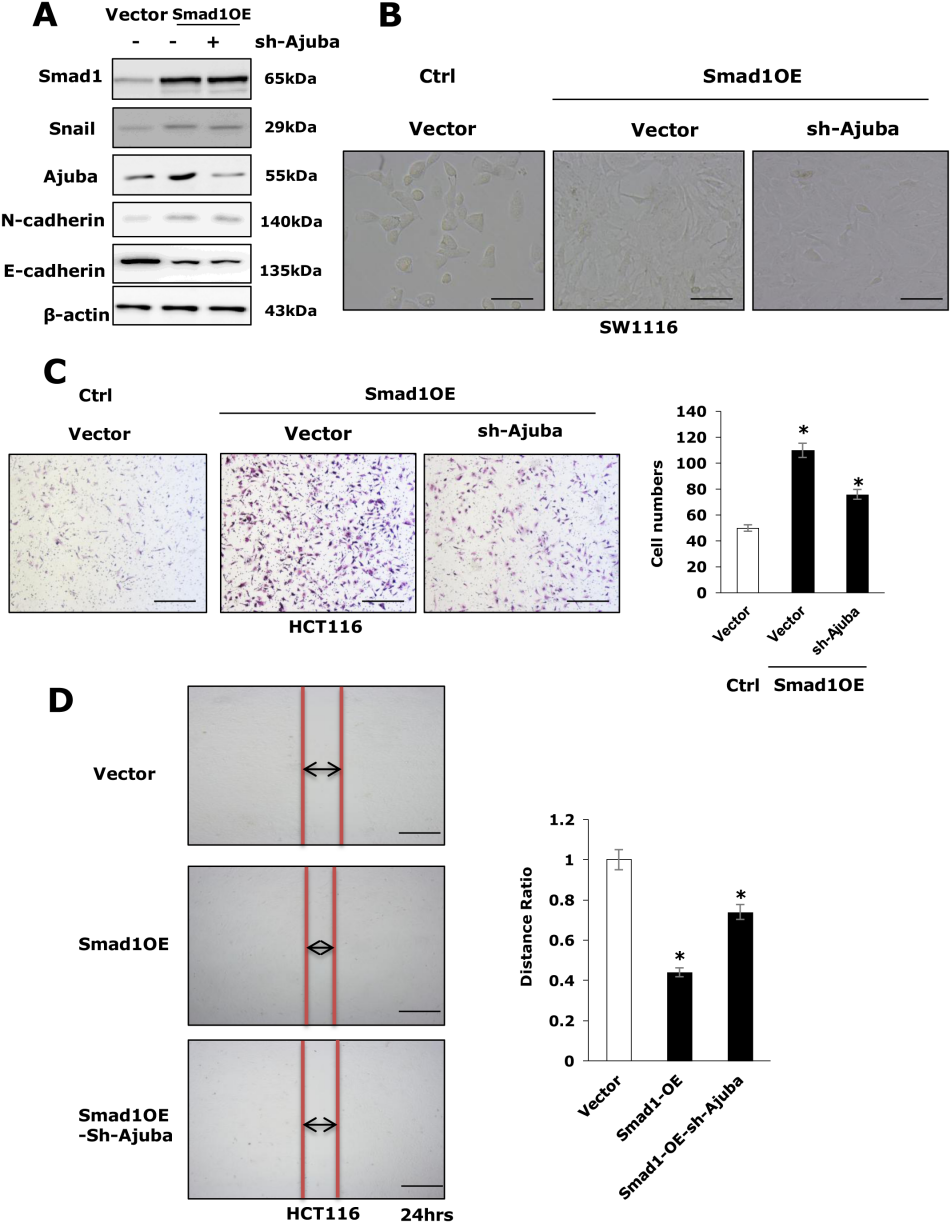
Ajuba was involved in cell migration and EMT induced by Smad1 deficiency in HCT116 cells **(A)** Depletion of Ajuba in Smad1 over expression HCT116 cells. Western blots show the Ajuba protein in HCT116 cells and the derivatives. **(B)** The cellular morphology of three cell lines (SW1116-Vector, SW1116-Smad1, and SW1116-Smad1-shAjuba) is shown. **(C)** Left panel: Transwell assays showed repressing of Ajuba in Smad1 overexpression cells decreases cell migration *in vivo* compared with Smad1 overexpression group. Right panel: Data were shown as mean± S.D. from three independent experiments, ^*^p<0.05(n=3). **(D)** Left panel: Scratch assays showed repressing of Ajuba in Smad1 overexpression cells decreases cell migration *in vivo* compared with Smad1 overexpression group. Right panel: Data were shown as mean± S.D. from three independent experiments, ^*^p<0.05(n=3).

To determine the importance of upregulation of Ajuba for Smad1 to regulate cell migration, we stably introduced vector containing shRNA specifically targeting Ajuba via viral infections into Smad1 overexpression HCT116 cells (Figure 4A and Supplementary Figure 1H). Remarkably, depletion of Ajuba in HCT116-Smad1 cells significantly changed cell morphology (Figure 4B), inhibited the cell migration examined by transwell and scratching assays (Figure 4C and 4D). These results, taken together, indicate that Ajuba is required for Smad1 to induce cell migration.

### Smad1 is positively correlated with Ajuba expression in colorectal cancer samples

**Figure 5:**
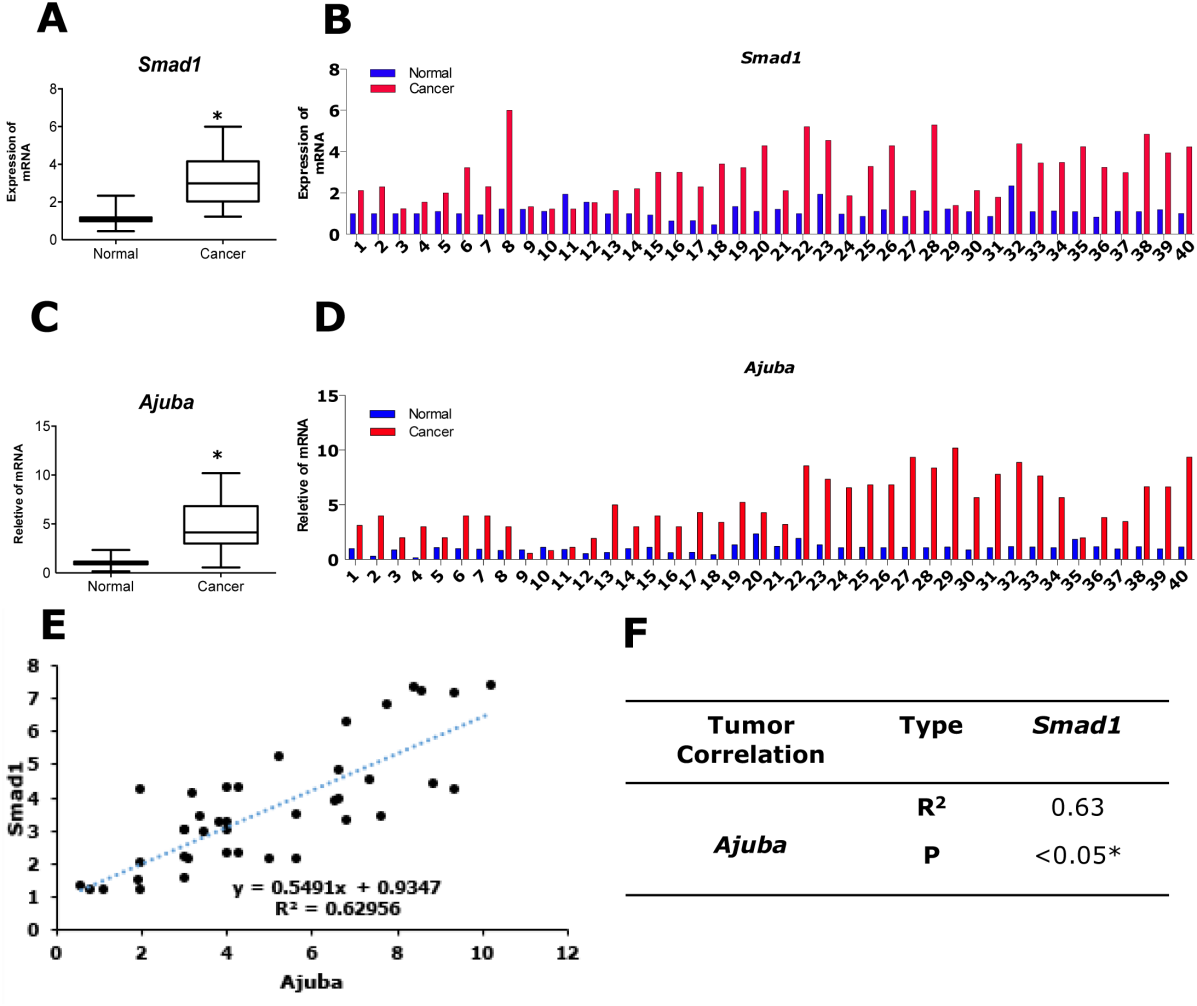
Clinical correlation of Smad1 in CRC patients **(A-B)** The mRNA expression of Ajuba in human CRC tissues and peri-cancerous normal tissues was compared by qPCR (n=40, paired t-test). Data were shown as mean± S.D. from three independent experiments, ^*^p<0.05(n=3). **(C-D)** The mRNA expression of Smad1 in human CRC tissues and peri-cancerous normal tissues was compared by qPCR (n=40, paired t-test). Data were shown as mean± S.D. from three independent experiments, ^*^p<0.05(n=3). **(E)** Correlation analysis shows that there exists a significant positive correlation between Ajuba and Smad1 in CRC samples. **(F)** Pearson's correlation analysis shows that there exists a significant correlation between Ajuba and Smad1 in CRC samples.

To evaluate the clinical relevance of Smad1 and Ajuba, we performed qRT-PCR assays on 40 paired CRC specimens. Consistent with previous observations, the expression of Smad1 was significantly higher in tumor compared with the para-tumor samples (Figure 5A and 5B). To examine the protein level of Ajuba and Smad1 in CRC specimens, we performed immunohistological chemistry (IHC) assays on tumour tissues (Supplementary Figure 3). Interestingly, Ajuba showed parallel expression pattern with Smad1 (Figure 5C and 5D). Pearson's correlation analysis showed that a significant positive correlation between the level of Smad1 and Ajuba in CRC samples (Figure 5E and 5F).

## DISCUSSION

**Figure 6:**
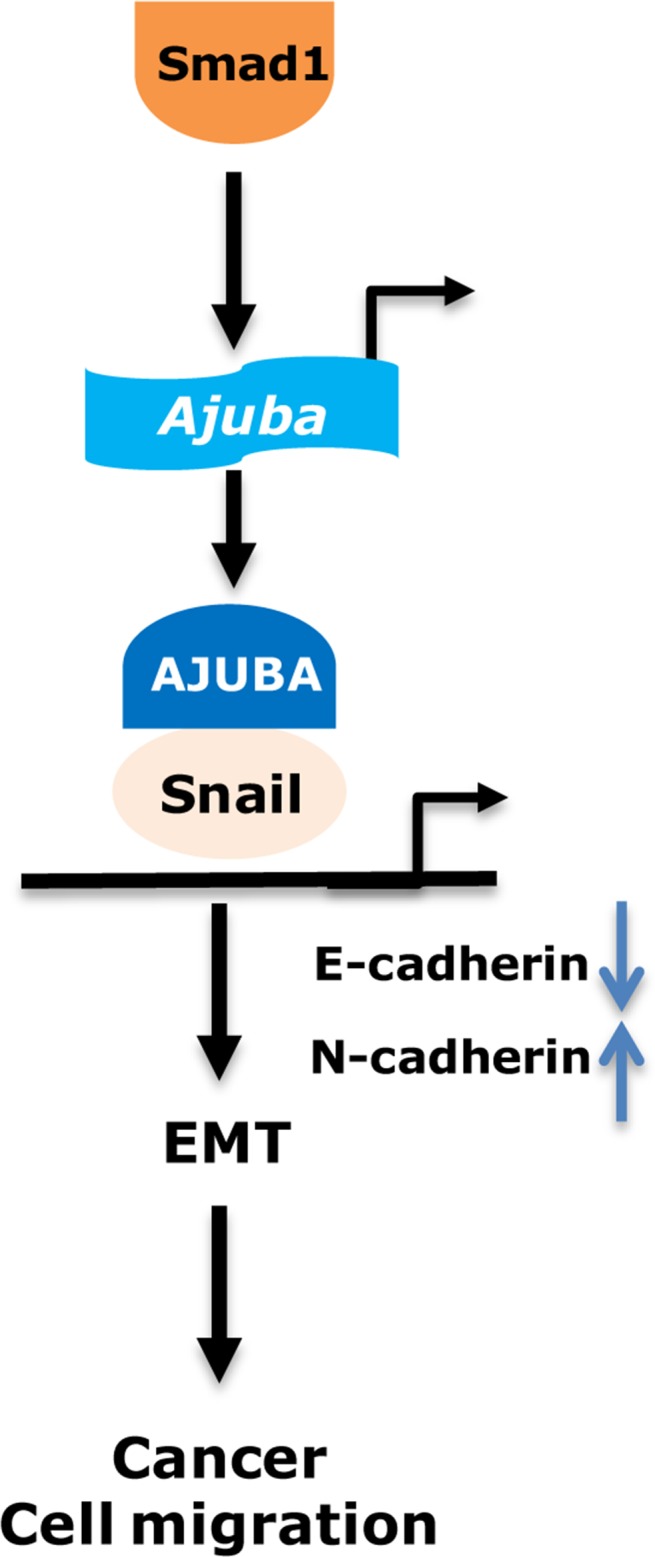
Working model show that Smad1 may contribute to the cell migration of CRC

Colorectal cancer is the third common cancer in men and the second in Women in worldwide [18]. However, the molecular mechanisms of tumorigenesis and migration of CRCs remain largely unclear. In this paper, we demonstrate that Smad1 promotes cell migration of colorectal cancer cells by upregulating Snail and Ajuba. Snail and Ajuba have been shown to form into a functional multi-protein complex to induce EMT and migration via transcriptional repression in various types of tumors (Figure 6). Moreover, the expression of Ajuba and Smad1 in colorectal cancer are positively correlated, suggesting that Smad1 and Ajuba may be potential therapeutic targets and prognostic factors for CRC.

The association of Smad1 with advanced cancer stage and migration are well documented. The expression of Smad1 in CRC patients have been reported by several groups in Oncomine database (https://www.oncomine.org). A series of studies also indicated that Smad1 is a critical inducer of the EMT process. PDGF-AA promotes mesenchymal stem cells migration via the BMP-Smad1/5/8-Twist1/Atf4 axis and Twist1 plays the role as a downstream factor of Smad1 [13]. However, our data showed that ectopic expression of Smad1 in HCT116 increases did not increase the expression of Twist1, instead, markedly induced Snail/Ajuba expression. Snail is well known as an critical EMT inducer and promotes metastatic and tumorigenic abilities in various types of cancers [11]. Ajuba functions as an obligate co-repressor for Snail and is essential for Snail-mediated breast cancer cell migration by recruiting PRMT5 to modulate histone modifications. A recent study also indicates that an elevated expression of Ajuba in CRC may contribute to the tumor metastasis by acting as a co-repressor of Snail [15]. Interestingly, a recent study showed that Smad1 as an upstream factor regulates Snail induced PI-3 kinase/Akt and Nanog expression [16]. How Smad1 transactivates the expression of Snail remains an interesting question and need to be explored further.

In summary, our findings here highlight an important role for Smad1-Ajuba/Snail signaling in CRC cell migration [14]. The elevated of Smad1 requires the induction of the Ajuba-Snail axis but not Twist1 to promote cell migration. Disruption of the Smad1/Ajuba/Snail axis may be potential targets for CRC therapeutics.

## MATERIALS AND METHODS

### Patients and tissue samples

A total of 40 colorectal adenocarcinoma specimens collected from patients, who received the operation at the Department of Digestive endoscopy center of the Tongren Hospital of Shanghai of China, were used in this study. All methods were carried out in accordance with relevant guidelines and regulations. All experimental protocols were approved by a shanghai jiaotong university licensing committee. Informed consent had been obtained from all patients and the project had been approved by the local Ethics Committee of Tongren Hospital of Shanghai of China.

### Western blot and antibodies

Cells were lysed in buffer containing 20 mmol/L Tris-HCl (pH 8.0), 150 mmol/L NaCl, 2.5 mmol/L EDTA, 0.5% NP40, 0.1 mmol/L phenylmethylsulfonylfluoride, and protease inhibitor cocktail. Antibodies for Smad1(Cell Signaling, #9743), Snail(Santa Cruz; sc-28199), N-cadherin (Cell Signaling, #4061), E-cadherin (Cell Signaling, #3195), Ajuba (Cell Signaling, #4897) and β-Actin (Proteintech, 600081-Ig) were purchased. The assay was carried out in triplicate.

### Real-time PCR analysis

Total RNA was extracted from cells with TRIzol reagent (Invitrogen) following the manufacturer's protocol. Complementary DNAs were synthesized with 2 μg of total RNA using iScriptcDNA Synthesis Kit (Fermentas). The detection and quantification of target mRNA were performed with real-time PCR. The assay was carried out in triplicate. Sequence of Primers for qRT-PCR:

Smad1: F:5’-ctcatgtcatttactgccgtgtgtg-3’ R: 5’-ATTCAGAAACGGTTCTTATTGTTG-3’

E-cadherin: F: 5’-TTGCTACTGGAACAGG GACAC-3’ R: 5’-CCCGTGTGTTAGTTCTGCTGT-3’

N-cadherin: F: 5’-TTATCCTTGTGCTGATGT TTGTG-3’ R: 5’-TCTTCTTCTCCTCCACCTTCTTC-3’

Snail F: 5’-TCCAGAGTTTACCTTCCAGCA-3’ R: 5’-CTTTCCCACTGTCCTACTCTG-3’

### Smad1 and Ajuba knockdown

Smad1 knockdown cells were determined with the siRNAs assay as previously described [14]. For transient Smad1 knockdown, siRNAs were used (Thermo Scientific and Santa Cruz Biotechnology, Inc.). For stable knockdown of Smad1, four siRNA hairpins expressed by a retrovirus vector (37928, 31427, 32418, and 30824, Dharmacon, Inc.) were tested. Two of them (37928 and 30824) could efficiently knock down Smad1 and were used further in this study. The plasmids of PLKO.1-shAjuba and shSmad1 were described previously [8].

### Cell culture, transfection and retroviral infection

HCT116 and 293T cells were obtained from the ATCC and were tested and authenticated by DNA typing. The cells were cultured in DMEM supplemented with 10% FBS, 2m ML- glutamine and penicillin (50 U/ml)/streptomycin (50 μg/ml) at 37°C under 5% CO_2_ in a humidified chamber. The transfection was performed using Lipofectamine 2000 (Invitrogen, Carlsbad, CA, USA) as described [7]. Supernatants containing viruses were packed from 293T cells. When growing to 60–80% confluence, HCT116 cells were infected with viral supernatants, and 5 μg/ml puromycin was added to select the stable cells.

### Transwell cell migration assays

Cells were harvested after serum-free starvation for 12 hrs, and were resuspended in plain DMEM media. Ten thousand cells were applied to 8-μm pore transwell filters (Corning). DMEM media containing 10% FBS were added to the bottom chamber as attractants. After incubation for 24 hrs, the nonmigrated cells at the top of the filter were removed and the migrated cells at the bottom of the filter were fixed with 4% paraformaldehyde and were stained with colloidal staining method. The number of migrating cells in each chamber was quantified by counting nine randomly chosen fields under ×20 magnification using a bright-field microscope. Each condition was performed in duplicate, and the average number of cells per field was represented. Experiments were repeated three times.

### Scratch assays

For the scratch assay, cells were grown to confluence in a 24-wells plate, and a “wounding” line was scratched into the cell monolayer with a sterile 200-mL pipette tip. The width of the wound was measured under a microscope at 0 and 24 hrs after the scratch to assess the migration ability of the cells.

### CCK-8 assays

Cells were aliquoted into a 96-well plate (2000 cells in 200 μl per well) and were incubated for 24 hrs, 48 hrs, 72 hrs and 96 hrs. Twenty microliters of cck-8 bromide solution were added at the indicated time point, and the aliquots were incubated for 1hrs. One hundred fifty microliters of dimethyl sulfoxide were replaced with 200ul DMEM that contained 10% FBS, and the aliquots were shaken for 10 minutes. Absorbance at 490 nm was measured to determine the number of viable cells in each well.

### Statistical analysis

Each experiment was repeated at least three times with comparable results. Results are expressed as mean± S.D. Statistical analysis was performed using Student's two tailed t-test exact test to compare *in vitro* and *in vivo* experiments, with P values < 0.05 considered statistically significant. Pearson's correlation analysis was used to determine the correlation of the expression levels of Smad1 using SPSS 13.0 software. All statistical tests were carried out using SPSS software (SPSS 13; SPSS Inc., Chicago, IL, USA).

## SUPPLEMENTARY MATERIALS FIGURES


