# Supplementary material for: Smad1 promotes colorectal cancer cell migration through Ajuba transactivation
Source: Oncotarget. 2017 Nov 30;8(66):110415–25. doi: 10.18632/oncotarget.22780 (PMC5746393; doi:10.18632/oncotarget.22780)
Supplement: Supplementary file 1 [file oncotarget-08-110415-s001.pdf]

Smad1 promotes colorectal cancer cell migration through Ajuba transactivation

SUPPLEMENTARY MATERIALS

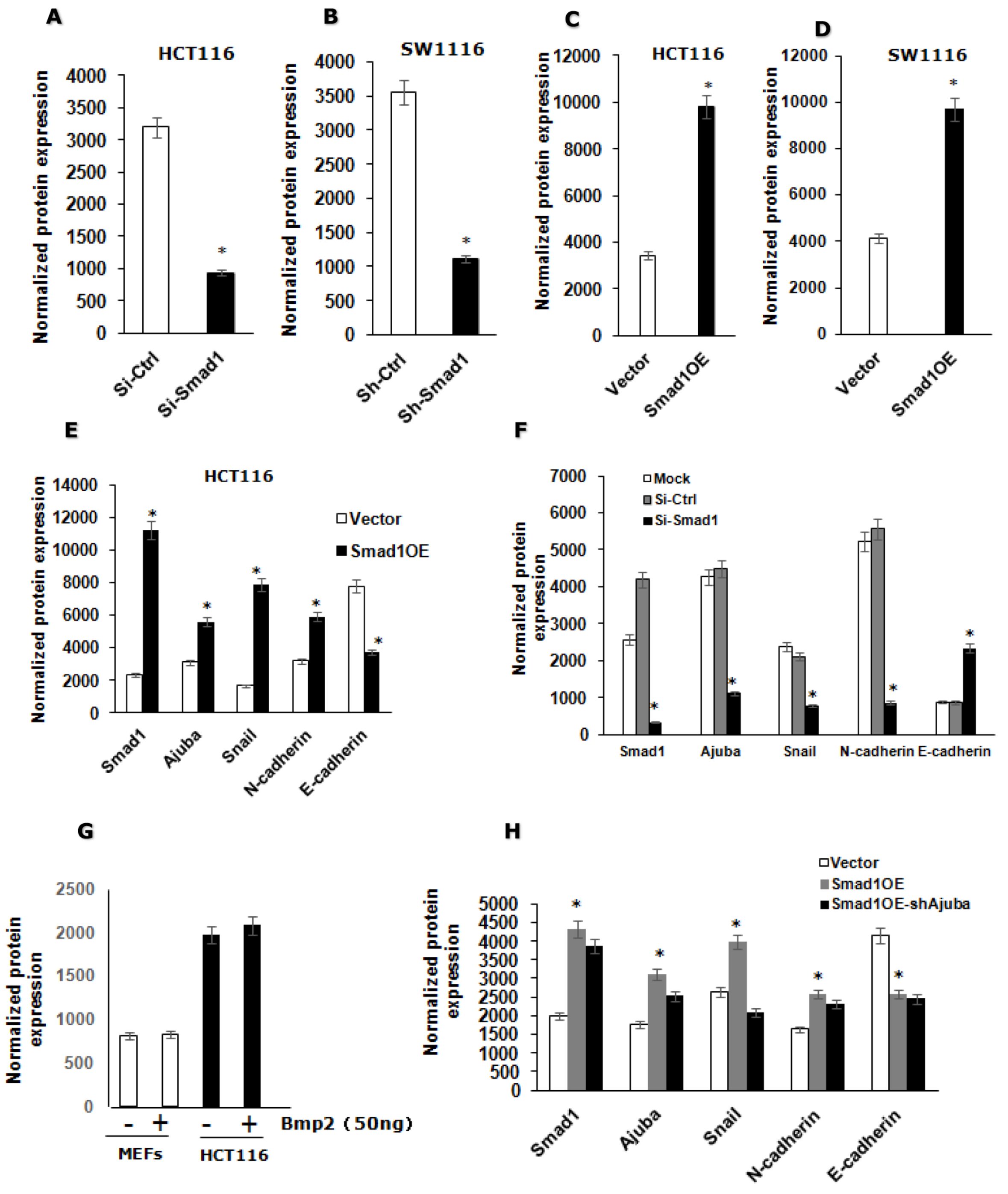

Supplementary Figure 1: The protein levels of Smad1, Ajuba, and EMT markers were analyzed by western blot. (A-H) panels show quantitation data. n = 3. $^{*}P < 0.05$.

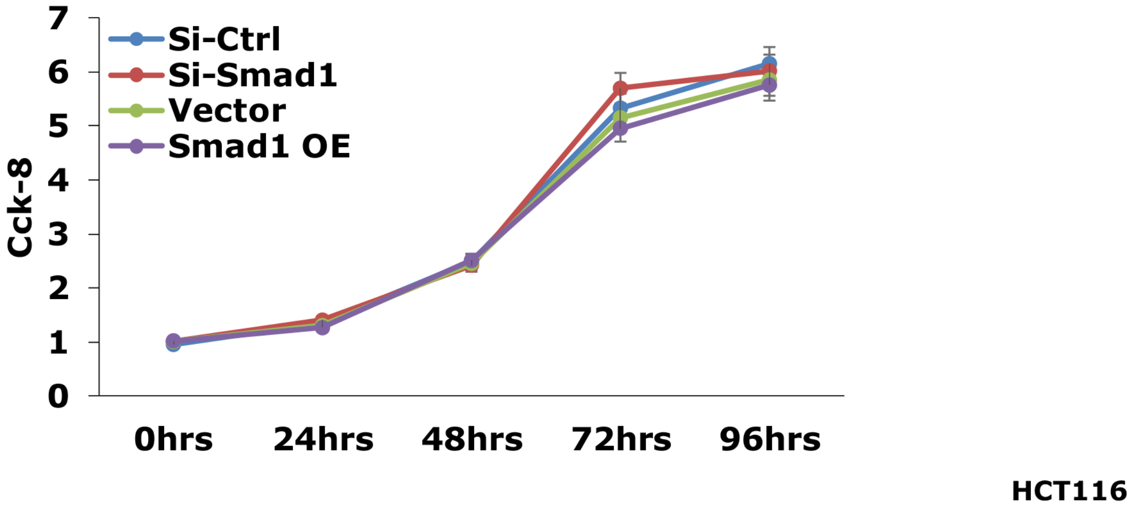

Supplementary Figure 2: Smad1 not affect the cell proliferation in HCT116. Cell growth was detected by cck-8 assay.

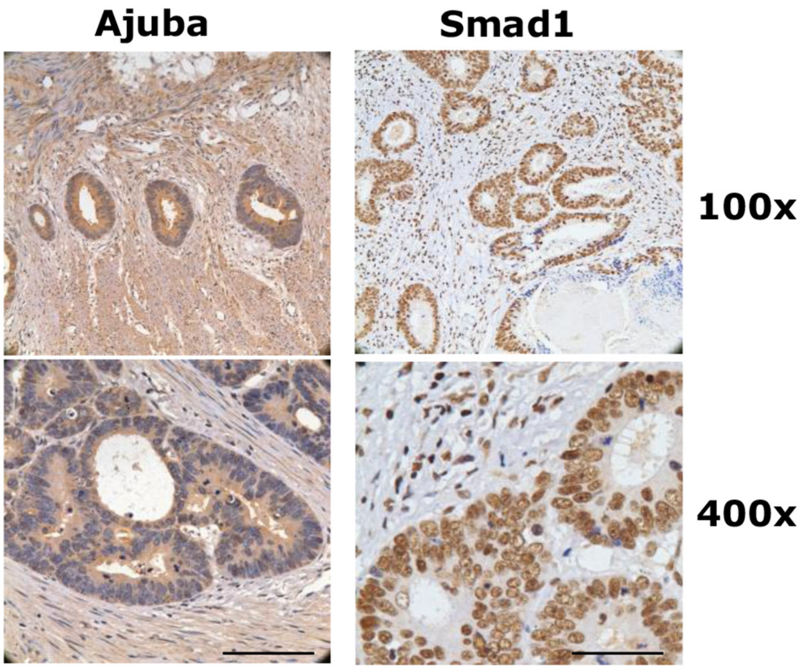

Supplementary Figure 3: Representative immunostaining images of Ajuba and Smad1 using serial sections of tumor samples.
